# LncRNA CASC9 promotes cell proliferation and invasion in osteosarcoma through targeting miR-874-3p/SOX12 axis

**DOI:** 10.1186/s13018-022-03340-w

**Published:** 2022-10-20

**Authors:** Haiyan Qiu, Di Yang, Xiaolin Li, Fabo Feng

**Affiliations:** 1 Department of Endocrinology, Affiliated Hangzhou First People’s Hospital, Zhejiang University School of Medicine, Hangzhou, 310004 China; 2 Center for Plastic and Reconstructive Surgery, Department of Orthopedics, Zhejiang Provincial People’s Hospital (Affiliated People’s Hospital, Hangzhou Medical College), No.158 Shangtang Road, Hangzhou, 310014 Zhejiang China

**Keywords:** Osteosarcoma, lncRNA, CASC9, miR-874-3p, SOX12

## Abstract

**Background:**

Osteosarcoma (OS) is a common primary malignant bone tumor. This study aimed to explore the biological role of long on-coding RNA (lncRNA) CASC9 and its regulatory mechanism in OC.

**Methods:**

The CASC9 expressions in OS cells and tissues were measured using qRT-PCR. The functional role of CASC9 in OC was studied using MTT assay, colony formation assay, transwell invasion assay, and xenograft tumor assay. In addition, the mechanism of CASC9 function was determined using luciferase reporter assay. Western blot was used to analyze protein expressions in our paper.

**Results:**

LncRNA CASC9 was found to be up-regulated in OS. Knockdown of CASC9 inhibited the proliferation and invasion of OS cells. Besides, miR-874-3p was identified as the target of CASC9, and SOX12 acted as a potential target of miR-874-3p. The down-regulation of miR-874-3p recovered the reduction in cell invasion and proliferation in vitro which were induced by CASC9 knockdown and delayed the tumor progression in vivo.

**Conclusion:**

LncRNA CASC9 promotes cell proliferation and invasion in OS via miR-874-3p/SOX12 axis. Our study might provide novel biomarkers and potential therapeutic targets for OS treatment.

## Introduction

Osteosarcoma (OS) is characterized by the proliferation of malignant tumor cells that can directly differentiate into bone or bone-like tissue [1]. OS has a high degree of malignancy and rapid progression, and it often metastases to the lung through blood in the early stage. OS accounts for 0.2% of the incidence of malignant tumors, and the overall incidence is about 3/1, 000, 000 [2]. Chemotherapy can significantly improve the prognosis of patients with non-metastatic OS, but chemotherapy efficacy is significantly lower in patients with combined metastasis, especially lung metastasis [3]. In recent years, radical tumor resection combined with neoadjuvant chemotherapy has been extensively used in OS clinical therapy [4]. Despite the advancement in diagnosis, chemotherapy, and surgical techniques, the prognosis of OS remains poor [5]. To develop effective targeted therapies for OS, extensive studies on the molecular mechanisms regulating the progression of OS are quite necessary.

With the continuous development of studies, the potential roles of non-coding RNAs (ncRNAs) have been reported in musculoskeletal conditions [6–9]. LncRNAs have been reported to serve as tumor-suppressive or oncogenic roles in the tumorigenesis and progression of a variety of malignancies, including OS [10]. LncRNAs, contains about 200 nucleotides, are distributed in both cytoplasm and nucleus. It is believed that lncRNAs in cytoplasm influence mRNA transcription and degradation [11] and can also help protein transport to the nucleus and activate gene expression [12]. Other lncRNAs are localized in the nucleus, which are involved in crucial molecular processes, including RNA processing, chromatin organization and transcription, and play an important role in genome structure [13, 14]. Cancer susceptibility candidate 9 (CASC9) is a conserved lncRNA with 1500 nucleotides (nt) in length. CASC9 was firstly reported in esophageal cancer as an oncogene [15] and was subsequently confirmed to be associated with the occurrence and development of several cancers, such as breast cancer [16], gastric cancer [17] and colorectal cancer [18]. Our preliminary experiment screened several lncRNAs from OS tissues and paired adjacent tissues by performing qRT-PCR, and identified lncRNA CASC9 was highly expressed in GC tissues. Nevertheless, there are few studies on the function and mechanism of CASC9 in OS progression.

In the current study, we aimed to explore the functional role of CASC9 in OS. Given its high expression in OC tissues, we hypothesized that CASC9 may play a tumor-promoting role in OC. Then, we investigated the expression of CASC9 in OC cell lines and detected the influences of CASC9 silencing on cell proliferation, and invasion in OS cells and in mouse model. In addition, the oncogenic mechanism of CASC9 in OS was also demonstrated.

## Materials and methods

### Tissue samples

A total of 30 paired of OS tumor tissues and adjacent non-tumor bone tissues were collected in our research. All OS patients involved in our study were enrolled from Department of Orthopedics from August 2015 to July 2020 at Zhejiang Provincial People’s Hospital, People’s Hospital of Hangzhou Medical College hospital. The patients underwent surgical resection and the specimens were collected. None of the patients received chemotherapy or radiotherapy prior to surgery. Liquid nitrogen was immediately used to snap-freeze these tissues. And then the tissues were placed at − 80 °C. This study was conducted according to the guidelines of the Declaration of Helsinki of 1975, as revised in 2013 and approved by the Ethics Committee of Zhejiang Provincial People’s Hospital, People’s Hospital of Hangzhou Medical College hospital (no.2020QT359). All patients or their advisers enrolled in our study signed the informed consent.

### Cell culture

Human OS cell lines, containing MG63, 143B, SW1353, U-2OS and HOS, and normal osteoblastic cell line (hFOB1.19), were all bought from Cell Bank of Shanghai Biology Institute (Chinese Academy of Science, Shanghai, China). DMEM, purchased from Invitrogen (Carlsbad, CA, U.S.A.), was added with penicillin/streptomycin (1%, Invitrogen), l-glutamine (2 mM) and fetal bovine serum (FBS) (10%, Invitrogen) and then, used to culture these OS cell lines at 37 °C in a 5% CO_2_ atmosphere. The hFOB1.19 cells were grown at a permissive temperature of 33.5 °C.

### Cell transfection

Small interfering RNA against lncRNA CASC9 (si-CASC9), negative control (si-NC), short hairpin CASC9 (shCASC9)/shNC, miR-NC/miR-874-3p mimics, and miR-874-3p inhibitor/inhibitor NC were all designed and synthesized by GenePharma (Shanghai, China). The sequences were as follows: si-CASC9, 5′-AUGAACAUCCACAAACACCAA-3′; si-NC, 5′-GAAUCCUACUUUCACAGCCAU-3′; shCASC9, 5ʹ-CCGGAAACUCUGGAAAUUCUUCCCUCGAGGGAAGAAUUUCCAGAGUUUUUUUUG-3ʹ; shNC, 5ʹ-CCGGACGUGACACGUUCGGAGAUUCUCGAGUUCUCCGAACGUGUCACGUUUUUUG-3ʹ; miR-874-3p mimics, 5′-CUGCCCUGGCCCGAGGGACCGA-3′; miR-NC, 5′-ACUACUGAGUGACAGUAGA-3′; miR-874-3p inhibitor, 5′-UCGGUCCCUCGGGCCAGGGCAG-3′; inhibitor NC, 5′-CAGUACUUUUGUGUAGUACAA-3′. SOX12 over-expression plasmid was constructed using the pcDNA3.1 vector (Invitrogen). Cell transfection was conducted by using Lipofectamine™ RNAiMAX (Invitrogen) according to the manufacturer's protocol. For transfection, U-2OS and 143B cells were seeded into a 6‑well plate at a density of 5 × 10^5^ cells/well and transfected with above plasmids or vectors (50 nM) until they reached 70% confluence. Subsequent experiments were performed 48 h post-transfection.

### RNA extraction and qRT-PCR analysis

A TRIzol reagent that was bought from Invitrogen was applied to isolate total RNA with the accordance of the manufacturer’s instructions. A PrimeScript RT Master Mix (TaKaRa, Dalian, China) was used to reversely transcribe the total RNA. PrimeScript RT reagent Kit and SYBR Premix Ex Taq (TaKaRa) and PrimeScript RT reagent Kit (TaKaRa) were employed to carry out the qRT-PCR according to the manufacturer’s instruction. GAPDH was acted as an internal control. The PCR primers used in our paper were as follows: GAPDH sense, 5′-GTCAACGGATTTGGTCTGTATT-3′ and reverse, 5′-AGTCTTCTGGGTGGCAGTGAT-3′; lncRNA CASC9 sense, 5′-GTTTAGAATGGAAGCAGCAAA-3′ and reverse, 5′-CAAAGCAATGGAAGCATGTA-3′; SOX12 sense, 5′-GTGAGCCAGGACGCAAC-3′ and reverse 5′-AACTGGGGAGCGAGGAG-3′; miR-874-3p sense 5′-CTGCCCTGGCCCGAGG-3′ and reverse 5′-GTTGTGGTTGGTTGGTTTGT-3′. After that, qPCR was carried out with ABI 7500 Real-Time PCR system (Applied Biosystems, Carlsbad, CA, USA). The expression fold changes were computed using 2^−ΔΔCT^ method.

### MTT assay

Following transfection, U-2OS and 143B cells (2 × 10^3^ cells per well) were seeded into 96-well plates. At 0, 24, 48, and 72 h after cell culture, 10 μL MTT solution (Solarbio, Beijing, China) was added to each well. Following incubation for 2 h, the medium was dumped. Then, a Microplate Reader (Bio-Rad, Hercules, CA, USA) was used to validate the optical density at 490 nm.

### Transwell assay

Matrigel-coated transwell chamber (Corning Incorporated, NY, USA) was used to examine the cell invasion following the user guide. The upper chamber with serum-free medium was added with transfected cell lines (5 × 10^3^). The lower chamber was added with complete culture medium. After 24 h of cell incubation, PFA (4%) was applied to fix the cell lines in the lower chambers, and the crystal violet solution was used to stain the invaded cell lines. Five fields were randomly selected for counting penetrating cells under microscope (Olympus, Tokyo, Japan).

### Colony formation assay

Cells were seeded in 6-well plates at a density of 500 cells/well, and culture medium was replaced every 3 days. After 14 days of culture in DMEM containing FBS, cell colonies were fixed with 4% paraformaldehyde for 10 min, stained with 0.1% crystal violet for 20 min, and photographed. The number of cell colonies with more than 50 cells was counted in each dish. A light microscope, provided by Olympus (Tokyo, Japan), was proceeded to counter the numbers of colony cells.

### Luciferase reporter assay

The target miRNA and mRNA of lncRNA CASC9 were predicted using miRDB and TargetScan, respectively. Mutant (Mut) and wild-type (Wt) and luciferase reporter vectors of lncRNA CASC9 containing miR-874-3p binding sites were harvested from GenePharma (Shanghai, China). SOX12-WT and SOX12-Mut luciferase reporter vectors were also purchased from GenePharma. Luciferase reporter vectors were transfected into HEK293T cells and then cotransfected with miR-NC/miR-874-3p mimics using Lipofectamine 2000. A dual-luciferase reporter assay system (Promega, Madison, WI, USA), was performed to validate the relative luciferase activity after transfection for 48 h.

### Western blot

A Radioimmunoprecipitation assay (RIPA) buffer (500 μL) was used to prepare the protein lysates. SDS–polyacrylamide gels (10%) were then used to resolve the proteins (60 μg per sample), and PVDF membranes were used to electrotransfer them. Nonfat dry milk (5%) was subsequently applied to block the membranes in Tris-buffered saline with Tween 20 (TBST) for 1 h at room temperature. Next, primary antibody was employed to incubate them overnight at 4 °C. After that, the HRP-conjugated secondary antibody (Santa Cruz, USA) was used to culture the membranes for 1 h at room temperature. Target proteins (Abcam, MA, USA) were probed using anti-SOX12 (ab54371, 1:2500) and anti-GAPDH (ab8245, 1:1000). Proteins were visualized using a gel imaging system (Bio-Rad) with enhanced chemiluminescence reagent (Pierce Technology, IL, USA). Protein bands were semi-quantified using ImageJ software (version 1.8.0; National Institutes of Health).

#### Tumor Xenograft assays

BALB/c nude mice, 6-week-old and 18–20 g weighed, were bought from Vital River (Beijing, China). The mice were kept in specific pathogen-free conditions. This research was approved by the Animal Ethics Committee of Zhejiang Provincial People’s Hospital, People’s Hospital of Hangzhou Medical College and complied with The Guide for the Care and Use of Laboratory Animals (no.20180006). Thereafter, the mice were divided into three groups ad libitum, including the shNC group, shCASC9 group or shCASC9 + miR-874 inh group (*n* = 6 per group). 143B cells (1 × 10^7^ cells/1 mL PBS) transfected with shNC, shCASC9 or shCASC9 + miR-874-3p inhibitor were subcutaneously injected into the right back of mice, respectively. Then, tumor size was recorded on the 7th, 14th, 21th and 28th day, and the tumor volume (*V*) was defined weekly using the following formula: *V* = [length (*L*) × width (*W*)^2^]/2. On the 28th day after injection, mice were anesthetized by an intraperitoneal injection of 50 mg/kg pentobarbital sodium and then, killed by cervical dislocation. The tumors were collected and weighed.

#### Immunohistochemistry (IHC)

After collecting xenograft tumor tissues, 4% paraformaldehyde was used to fix the tissues for 2 days, and then, paraffin was used to embed them. The sections were blocked in 1% BSA for 20 min and subsequently cultured with primary antibody against Ki67 (1:20, ab21700, Abcam) at 4 °C overnight. Next, A HSP-labeled Goat Anti-Rabbit IgG H&L (1:500, ab205718, Abcam) was applied to incubate the sections for 30 min at room temperature. Later, hematoxylin was used to counterstain the slides. The images were taken under microscopy (Nikon Microsystems, Tokyo, Japan).

### Statistical analysis

All experiments were performed in triplicates and were repeated at least three times. SPSS 20.0 software (IL, USA) was used for statistical analyses. Data were presented as mean ± standard deviation (SD). Correlation among lncRNA CASC9, miR-874-3p and SOX12 expressions in OS tumor tissues was assessed via Pearson’s correlation analysis. Student’s t-test and one-way analysis of variance (ANOVA) followed by Dunnett’s multiple comparison were performed to analyze the differences among different groups. The relationship between CASC9 and clinicopathological features of the 30 cases of OS patients was determined via *χ*^2^ test. Kaplan–Meier method was performed to carry out the survival analysis. *P* < 0.05 was considered statistically significant.

## Results

### LncRNA CASC9 was highly expressed in OS tissues and cell lines

**Fig. 1:**
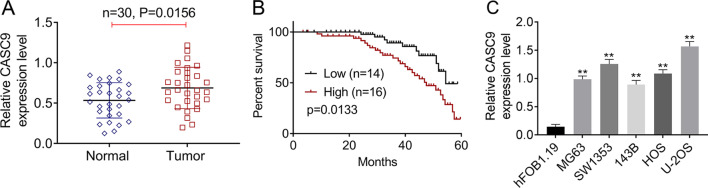
LncRNA CASC9 was over-expressed in OS tissues and cell lines. **A** The expression levels of lncRNA CASC9 in 30 pairs of OS tumor tissues and normal tissues were detected using qRT-PCR. **B** Kaplan–Meier method was performed to carry out the survival analysis. **C** The expression levels of lncRNA CASC9 in human OS cell lines (MG63, SW1353, 143B, HOS and U-2OS) and human normal osteoblastic cell line hFOB1.19 were assessed using qRT-PCR. **represented *P* < 0.01

First of all, OS tumor tissues (*n* = 30) and matched adjacent non-tumor tissues (*n* = 30) were collected in our research, and then, qRT-PCR was proceeded to detect the expressions of CASC9 in these tissues. Data from qRT-PCR showed that the expression of CASC9 in OS tumor groups was memorably higher than that in the normal groups (Fig. 1A, *P* = 0.0156). And, we found that the higher expression of CASC9 indicated the less percent survival of OS patients via Kaplan–Meier method (*P* = 0.0133, Fig. 1B). Besides, the expression of CASC9 in OS tumor tissues was associated with tumor size and TMN stage of OS patients (Table 1). In addition, the CASC9 expressions in human OS cell lines (MG63, 143B, SW1353, U-2OS and HOS), and human normal osteoblastic cell line hFOB1.19 were also assessed by qRT-PCR. Data from qRT-PCR showed that CASC9 was significantly over-expressed in OS cell lines compared to that in hFOB1.19 cell lines (*P* < 0.01, Fig. 3C). Among these five OS cell lines used in our research, CASC9 showed a highest expression in U-2OS cell line as well as a lowest expression in 143B cell line. Therefore, 143B and U-2OS cell lines were selected to proceed following analyses. These results confirmed that lncRNA CASC9 was highly expressed in OS and foreboded poor prognosis of OS patients.

**Table 1:** The correlation between CASC9 expression and OS clinical pathology

Characteristics	Number of patients	CASC9 Low expression (≤ median)	CASC9 High expression (> median)	*P* value
Number	30	14	16	
Ages(years)				0.358
< 18	15	8	7	
≥ 18	15	6	9	
Gender				0.509
Female	14	7	7	
Male	16	7	9	
Tumor size				0.035
≤ 2 cm	13	9	4	
> 2 cm	17	5	12	
TMN stage				0.014
I–II	14	10	4	
III–IV	16	4	12	
Lymphatic metastasis				0.642
Yes	15	7	8	
No	15	7	8	

### LncRNACASC9 knockdown inhibited the OS cell proliferation and invasion

**Fig. 2:**
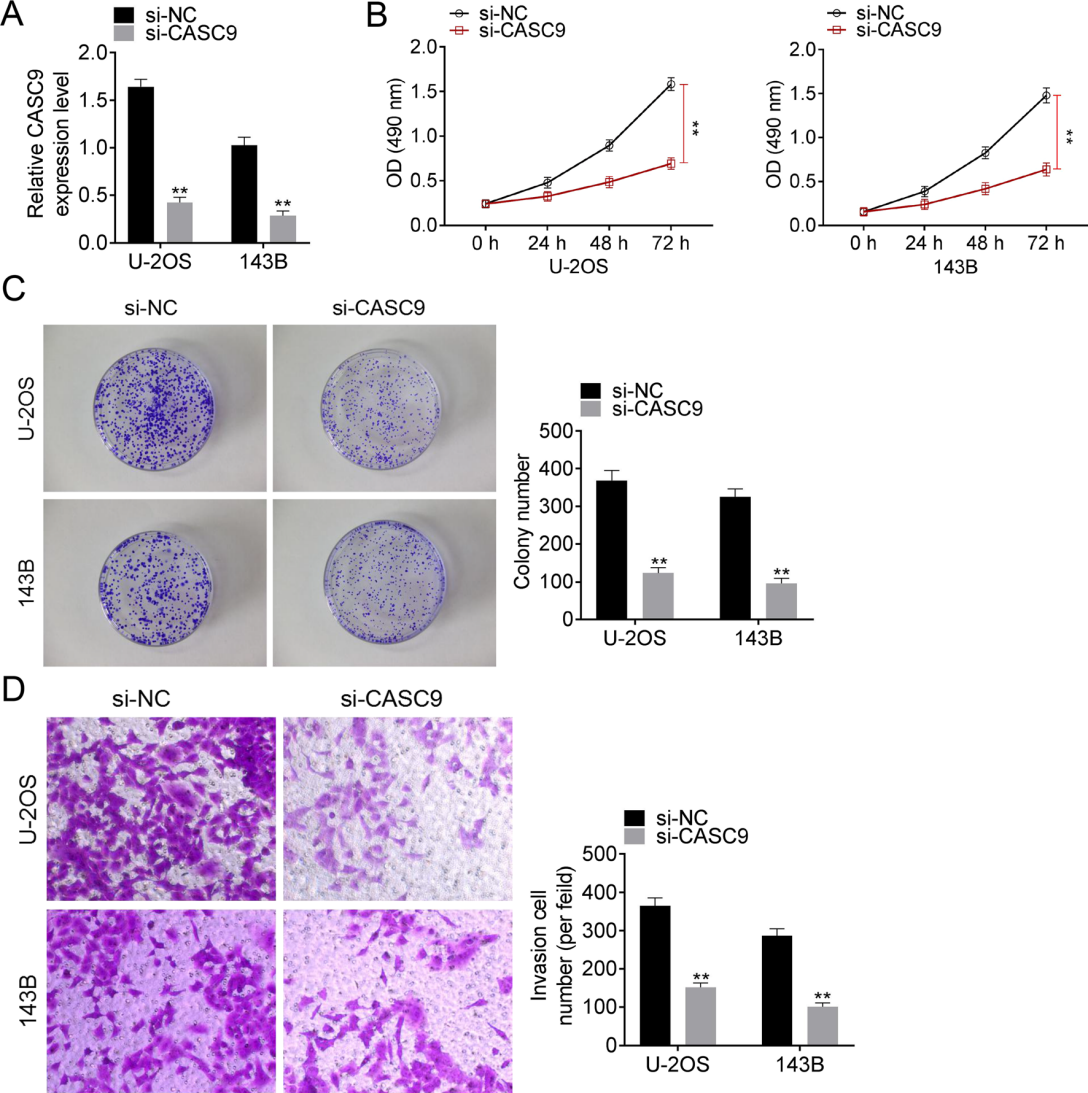
LncRNA CASC9 knockdown inhibited cell proliferation, colony formation and invasion in OS. **A** The expression of lncRNA CASC9 was detected using qRT-PCR. **B** The cell proliferation was analyzed using MTT assay. **C** The cell colony formation activity was examined using cell colony formation assay. **D** The cell invasion was assessed using transwell assay. **represented *P* < 0.01

In order to understand the effect of CASC9 on OS cell lines, the expression of CASC9 in 143B and U-2OS cell lines was disrupted by si-CASC9 construction and transfection. Data from qRT-PCR exhibited that CASC9 was significantly knocked down in si-CASC9 groups (Fig. 2A, *P* < 0.01). Then, MTT assay was operated to examine the cell proliferation of 143B and U-2OS that transfected with si-NC/si-CASC9 and the result of MTT assay is showed as Fig. 2B. The data showed that si-CASC9 transfection decreased CASC9 expressions both in 143B and U-2OS cells when compared to si-NC transfection group (Fig. 2B, *P* < 0.01). Colony formation assay was subsequently carried out to analyze the colony ability of U-2OS and 143B cell line. Similarly, the colony cell number of si-CASC9 groups was markedly lower than that of si-NC groups (Fig. 2C, *P* < 0.01). In addition, the cell invasion of 143B and U-2OS cell line that treated with si-CASC9 or si-NC transfection was assessed using transwell assay. It was confirmed that si-CASC9 transfection remarkably reduced invasion cell number compared to si-NC transfection both in 143B and U-2OS cell line (Fig. 2D, *P* < 0.01). The above data revealed that the silence of lncRNA CASC9 inhibited cell proliferation, colony formation and invasion in OS.

### LncRNA CASC9 directly targeted to miR-874-3p in OS

**Fig. 3:**
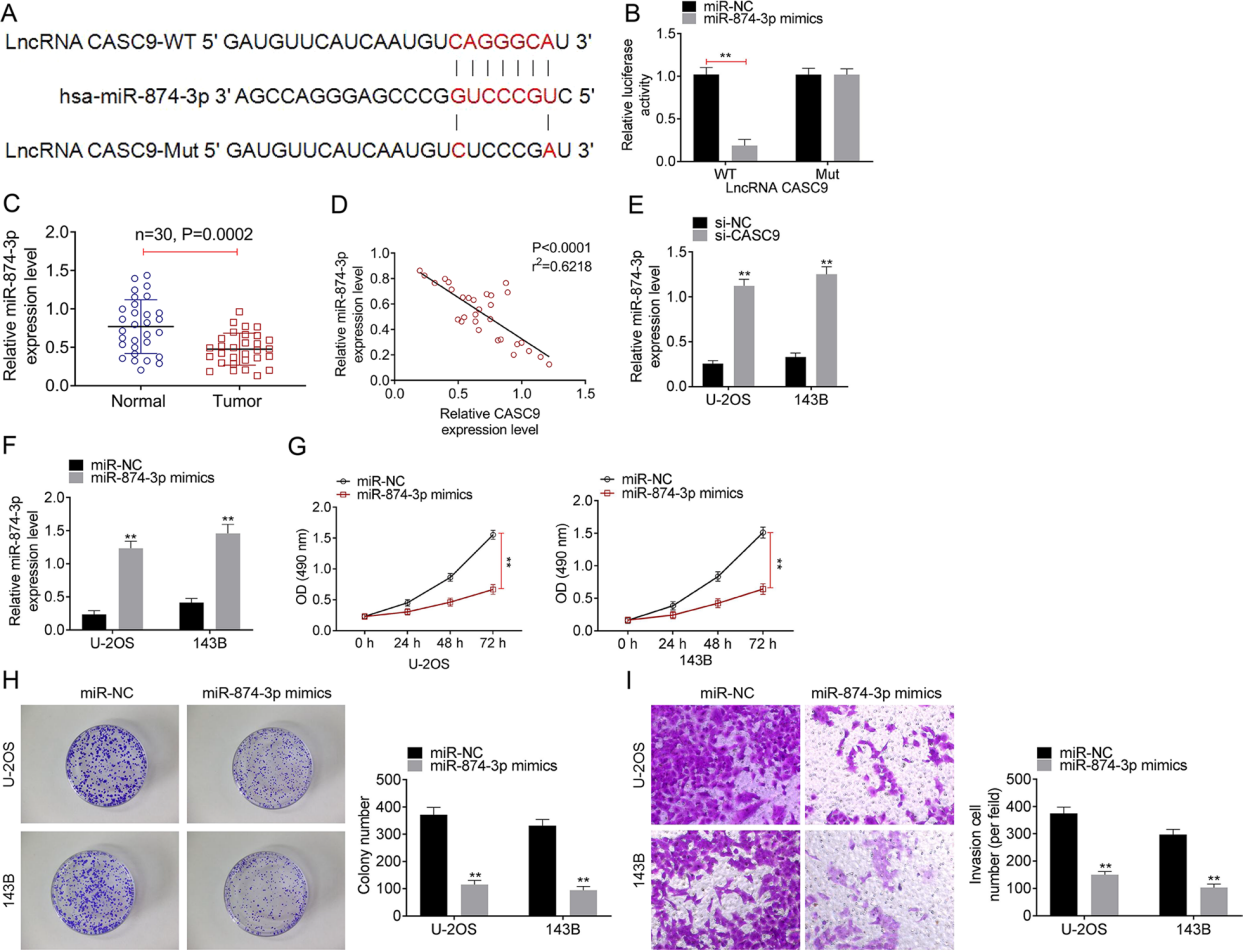
LncRNA CASC9 directly sponging miR-874-3p in OS. **A** The binding sites of CASC9 on miR-874-3p were predicted by miRDB. **B** The binding of CASC9 and miR-874-3p was verified by luciferase reporter assay. **C** The expression levels of miR-874-3p in 30 pairs of OS tumor tissues and normal tissues were detected using qRT-PCR. **D** The relationship between lncRNA CASC9 expression and miR-874-3p expression was calculated using Pearson’s *χ*^2^ test. **E**, **F** The expression of miR-874-3p was assessed using qRT-PCR. **G** The cell proliferation was analyzed using MTT assay. **H** The cell colony formation activity was examined using cell colony formation assay. **I** The cell invasion was assessed using transwell assay. **represented *P* < 0.01

**Fig. 4:**
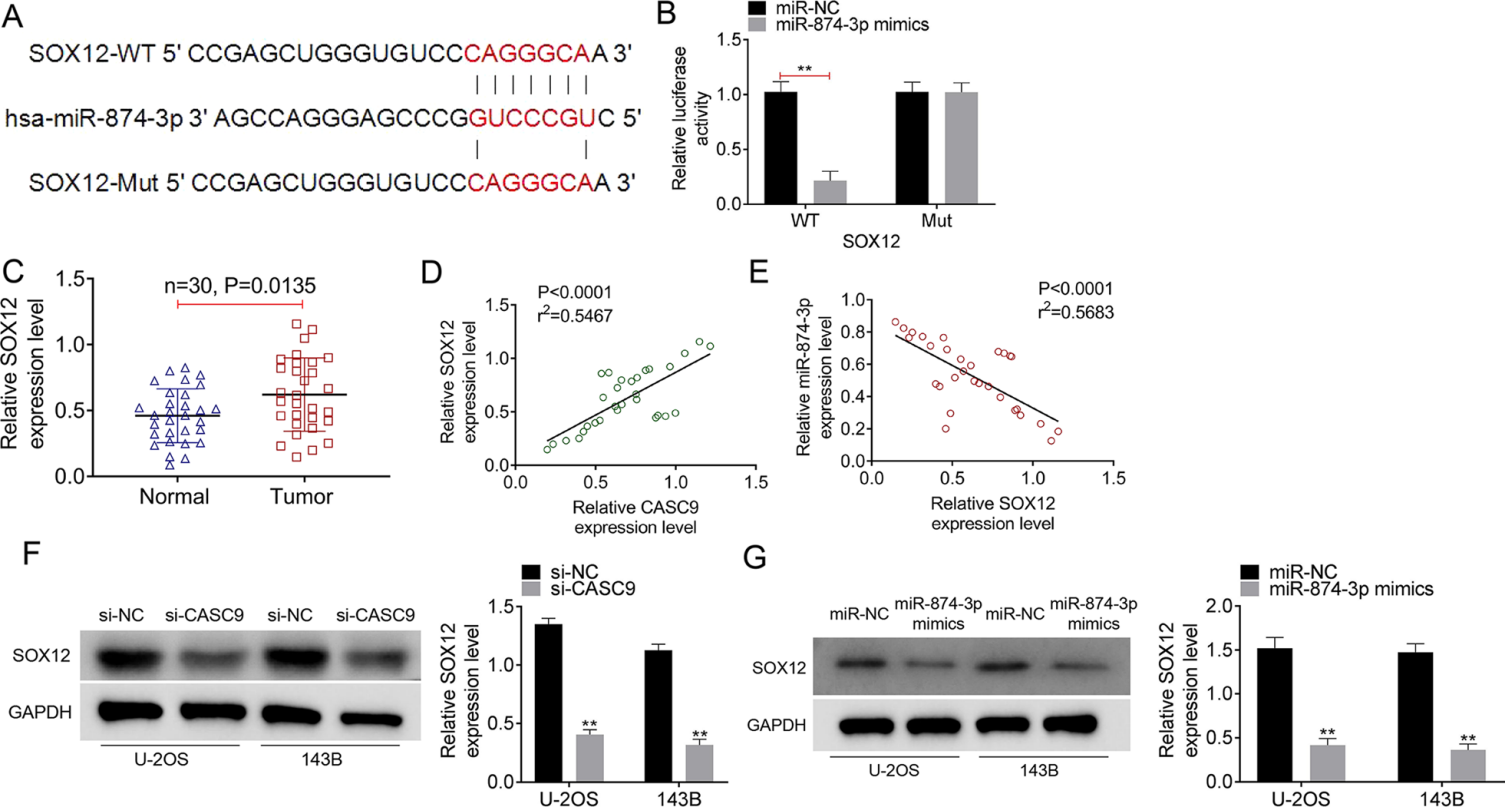
LncRNA CASC9/miR-874-3p directly targeted SOX12 in OS. **A** The binding sites of SOX12 on miR-874-3p were predicted by TargetScan. **B** The binding of SOX12 and miR-874-3p was verified by luciferase reporter assay. **C** The expression levels of SOX12 in 30 pairs of OS tumor tissues and normal tissues were detected using qRT-PCR. **D** The relationship between lncRNA CASC9 expression and SOX12 expression was calculated using Pearson’s *χ*^2^ test. **E** The relationship between SOX12 expression and miR-874-3p expression was calculated using Pearson's* χ*^2^ test. **F**, **G** The expression of SOX12 was assessed using Western blot. **represented *P* < 0.01

We predicted that miR-874-3p served as the target miRNA of lncRNA CASC9 by miRDB. To verify the prediction, CASC9-Mut and CASC9-WT luciferase reporter plasmids were established (Fig. 3A), and luciferase reporter assay was proceeded. As shown as Fig. 3B, miR-874-3p mimics transfection significantly cut down the relative luciferase activity after being transfected with CASC9-WT (*P* < 0.01). Then, the miR-874-3p expressions in OS tumor tissues and adjacent normal tissues were detected using qRT-PCR. Data from qRT-PCR displayed that the expression of miR-874-3p was observably decreased in OS tumor tissues compared with that in normal tissues (Fig. 3C, *P* = 0.0002). And, Fig. 3D showed that CASC9 expression level was negatively correlated with miR-874-3p expression level in OS tissues (*P* < 0.0001). Similarly, we confirmed that miR-874-3p was notably up-regulated with si-CASC9 transfection in U-2OS and 143B cell lines compared to si-NC transfection by qRT-PCR (Fig. 4E, *P* < 0.01). Data from qRT-PCR also showed that when the U-2OS and 143B cell lines was transfected miR-874-3p mimics plasmids, the expression level of miR-874-3p was dramatically enhanced (Fig. 4F, *P* < 0.01). Then, the cell proliferation ability of U-2OS and 143B cell lines that were treated with miR-874-3p mimics or miR-NC was detected through MTT assay, and the results of MTT assay are showed as Fig. 3G. When the expression of miR-874-3p was increased in the U-2OS and 143B cell lines, the cell proliferation ability was decreased (Fig. 3G, *P* < 0.01). Besides, colony formation assay was performed to examine the colony viability of U-2OS and 143B cell lines with miR-874-3p mimics/miR-NC transfection. Figure 3H revealed that miR-874-3p mimics transfection memorably repressed cell colony formation in OS cell lines compared to that miR-NC groups (*P* < 0.01). In addition, the cell invasion was tested using transwell assay and the results from transwell assay defined that the up-regulation of miR-874-3p in OS cell lines remarkably suppressed cell invasion ability (Fig. 3I, *P* < 0.01). These results proved that lncRNA CASC9 acted as a sponge of miR-874-3p in OS, and the expression of CASC9 in OS tissues and cell lines was negatively related to the expression of miR-874-3p. And, the over-expression of miR-874-3p could inhibited cell proliferation, colony formation and invasion in OS cell lines.

### LncRNA CASC9/miR-874-3p directly targeted to SOX12 in OS

To further explore the function of lncRNA CASC9 in OS, we predicted that SOX12 acted as the downstream mRNA of miR-874-3p by Targetscan and the binding sites are showed as Fig. 4A. SOX12-Mut and SOX12-WT were established to verify the prediction. As shown as Fig. 4B, the relative luciferase activity of miR-874-3p mimics group was significantly inhibited after being SOX12-WT transfection (*P* < 0.01). Then, the SOX12 expressions in 30 paired of OS tumor tissues and matched normal tissues were assessed by qRT-PCR, and Fig. 4C showed that SOX12 was markedly over-expressed in OS tumor tissues compared to that in normal tissues (*P* = 0.0135). Data from Pearson’s correlation analysis revealed that SOX12 expression was positively correlated with CASC9 expression and negatively correlated with miR-874-3p expression in OS tissues (Fig. 4D, E). In addition, the results of Western blot displayed that in U-2OS and 143B cell lines, the down-regulation of CASC9 significantly inhibited SOX12 expression (Fig. 4F, *P* < 0.01) while the up-regulation of miR-874-3p dramatically promoted SOX12 expression (Fig. 4G, *P* < 0.01). These data demonstrated that lncRNA CASC9 modulated the miR-874-3p expression, thereby influencing the SOX12 expression in OS cells.

### LncRNA CASC9-regulated cell proliferation and invasion through miR-874-3p/SOX12 axis in OS

**Fig. 5:**
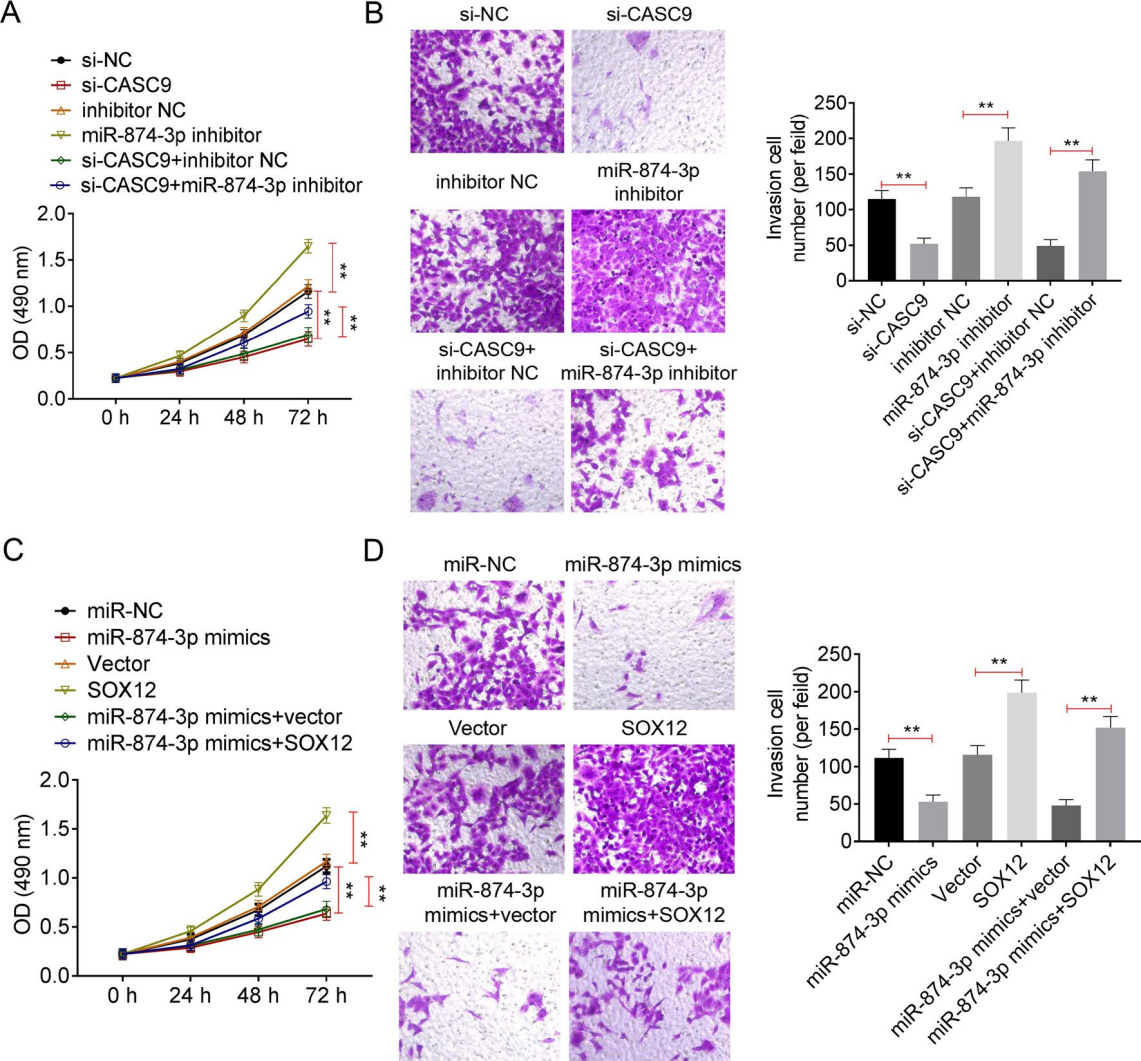
LncRNA CASC9 regulated cell proliferation and invasion by regulating miR-874-3p/SOX12 in OS. **A** The cell proliferation was detected using MTT assay. **B** The cell invasion was examined using transwell assay. **C** The cell proliferation was detected using MTT assay. **D** The cell invasion was examined using transwell assay. *represented *P* < 0.01

To further identify our conclusion, the U-2OS cell lines were divided into 6 groups (si-NC, si-CASC9, inhibitor NC, miR-874-3p inhibitor, si-CASC9 + inhibitor NC and si-CASC9 + miR-874-3p inhibitor) according to different treatments. Then, the cell proliferation and invasion were, respectively, detected using MTT assay and transwell assay. As shown as Fig. 5A, the inhibition of miR-874-3p significantly increased the reduction in cell proliferation of U-2OS cell lines caused by si-CASC9 transfection (*P* < 0.01). And, Fig. 5B showed that miR-874-3p inhibitor transfection effectively recovered the decrease in U-2OS invasion cell number that induced by CASC9 down-regulating (*P* < 0.01). Then, U-2OS cell lines were, respectively, transfected with miR-NC, miR-874-3p mimics, Vector, SOX12, miR-874-3p mimics + vector or miR-874-3p + SOX12 plasmids. Data from MTT assay and transwell assay, respectively, exhibited that SOX12 over-expression enhanced the reduction in U-2OS cell proliferation and invasion which were caused by miR-874-3p up-regulation (Fig. 5B, *P* < 0.01). In summary, above data demonstrated that lncRNA CASC9 promoted cell proliferation and invasion through regulating miR-874-3p/SOX12 in OS.

### *LncRNA CASC9 knockdown inhibited OS tumor growth *via* regulating miR-874-3p *in vivo

**Fig. 6:**
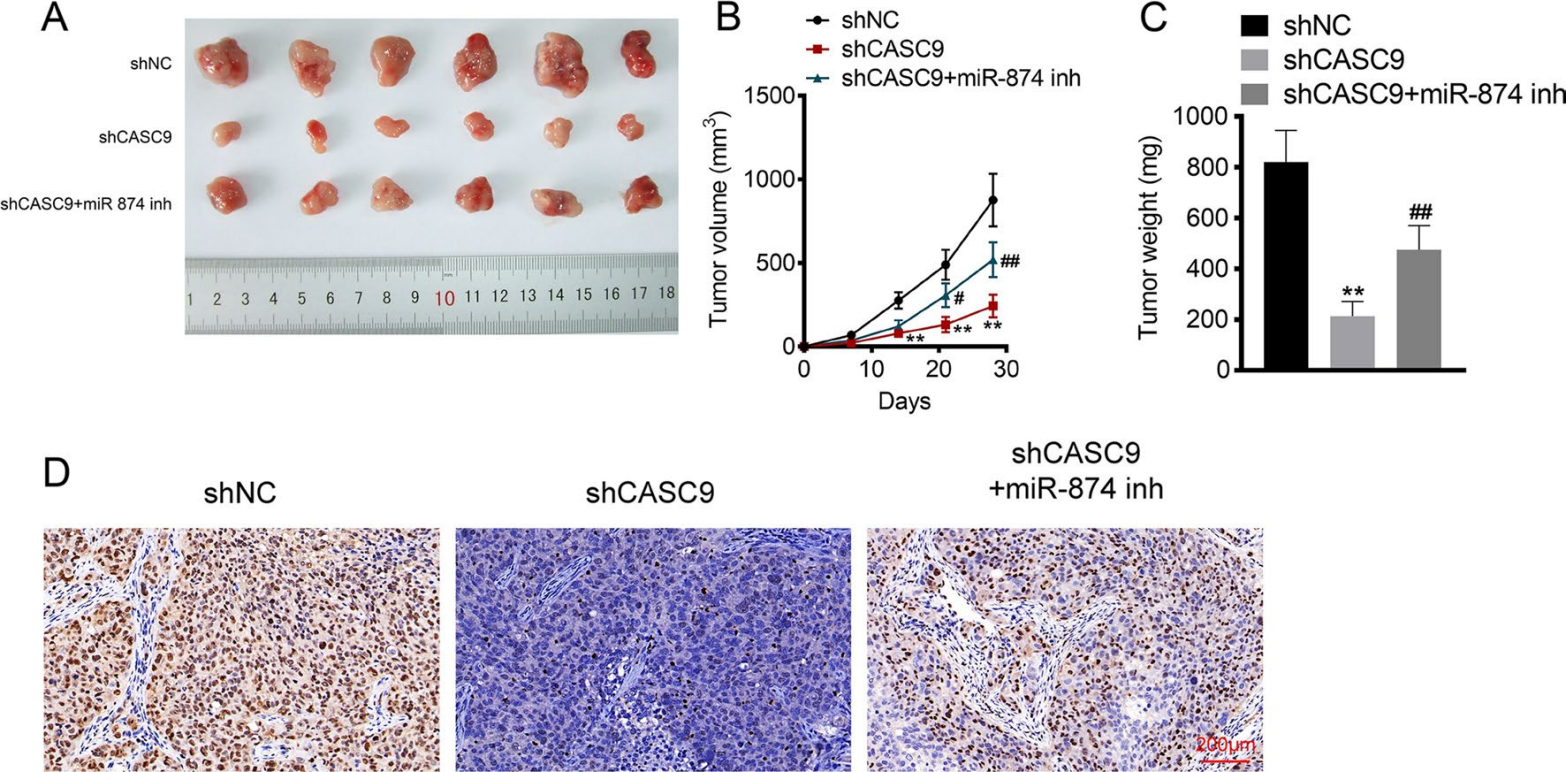
LncRNA CASC9 knockdown inhibited OS tumor growth via regulating miR-874-3p in vivo. **A** The tumor size of OS mice model was tested after the mice were killed. **B** The tumor volume of OS mice model was calculated and recorded weekly. **C** The tumor weight of OS mice model was weighed after the mice were killed. **D** The expression of Ki67 in mice tumor tissues was assessed using IHC assay. **P < 0.01 versus shNC group, ##*P* < 0.01 versus shCASC9 group

To evaluate the influences of CASC9 on OS in vivo, OS mice model was established and divided into three groups (shNC, shCASC9, shCASC9 + miR-874 inh) based on different treatments. The knockdown of CASC9 inhibited OS tumor volume, size and weight in vivo (*P* < 0.01, Fig. 6A–C). However, these effects were recovered when miR-874 was silenced at the same time. Then, IHC was performed to measure the expression of Ki-67. Data from IHC exhibited that the expression of Ki-67 was notably inhibited with shCASC9 transfection and enhanced with the co-transfection of shCASC9 and miR-874 inh (Fig. 6D). The above results elucidated that the silence of CASC9 could suppress OS tumor growth and proliferation through regulating miR-874 in vivo.

## Discussion

Given that the prognosis of OS is unsatisfactory, the development of novel strategies in its therapy is still of great significance. Increasing evidences are improving the understanding that lncRNAs play crucial roles in the pathogenesis and development of many cancers [19, 20]. Therefore, it is important to elucidate the molecular mechanism of prognosis-related lncRNAs and explore potential therapeutic targets. This study focused on the mechanistic involvement of CASC9 in OS. First, it was found that CASC9 silencing inhibited the proliferation and invasion of U-2OS and 143B cells. Moreover, the inhibition of miR-874-3p remarkably recovered cell proliferation and invasion in CASC9-silenced cells. Additionally, this study found that SOX12 is the target of miR-874-3p. SOX12 expression was increased in OS tissues; however, it was inhibited in OS cells by silencing CASC9 or over-expressing miR-874-3p. The results revealed an abundance of CASC9 through sponging miR-874-3p and positive regulation of SOX12 protein level, consequently contributing to the proliferation and invasion of OS cells. To our knowledge, this is the first study that reported the essential regulatory role of CASC9 and its underlying mechanism in OS progression.

The role of a small number of tumor-suppressive and oncogenic lncRNAs in OS has been elucidated. For example, Wang et al. [21] proved that lncRNA SNHG16 was up-regulated in OS, and the down-regulation of SNHG16 declined cell proliferation, invasion and migration ability via inversely regulating miR-1301 expression level in OS cell lines. Guo et al. [22] indicated that lncRNA steroid receptor RNA activator 1 (SRA1) acted as an antitumor role in OS, it inhibited cell activities and enhanced cell apoptosis by targeting miR-208a. Yu et al. [23] reported that lncRNA Taurine up-regulated gene 1 (TUG1) was abnormally expressed in OS and it accelerated cell metastasis through modulating HIF-1α via sponging miR-143-5p. However, the functional role of lncRNA CASC9 played in OS was still unclear. LncRNA CASC9 was defined as an oncogene in various cancers, such as breast cancer [16], colorectal cancer (CRC) [18], non-small cell lung cancer (NSCLC) [24], and esophageal squamous cell carcinoma [25] but never in OS. In the present study, we firstly proved that CASC9 was over-expressed in OS. Specifically, the overall survival of OS patients with high CASC9 levels was shorter than that of those with low CASC9 levels. CASC9 knockdown led to restricted proliferation and invasiveness in OS cells. Additionally, CASC9 down-regulation impeded tumor growth in vivo. Our results may provide a new biomarker for OS diagnosis and treatment.

Next, we explored the potential mechanism of lncRNA CASC9 in OS. One of the main functions of lncRNAs was acting as ceRNAs to regulate miRNA expressions [26, 27]. In our current study, miR-874-3p was predicted by miRDB as a potential target of CASC9. MiR-874-3p plays a tumor-suppressive role in different types of cancers, including ovarian cancer [28], hepatocellular carcinoma [29] and OS [30]. In OS, miR-874-3p is down-regulated and targets RGS4 to suppress cell proliferation and migration [30]. Consistently, our study also showed the down-regulation of miR-874-3p in OS and its inhibitory effects on OS cell proliferation and invasion. Herein, we established the ceRNA network that linked CASC9 with miR-874-3p. Notably, miR-874-3p absence could reverse the effect of silencing CASC9 on the proliferation and invasion of OS cells. Moreover, in vivo experiments showed that the suppressive effect of CASC9 silencing on tumor growth was partly blocked by inhibiting miR-874-3p, suggesting that the effect of CASC9 on OS was partly exerted by regulating the expression of miR-874-3p.

However, the molecular mechanisms that underlie the tumor suppressive role of miR-874-3p remain unknown. In the present study, the putative binding sites of the miR-874-3p and SOX12 were predicted via bioinformatic analysis and verified by luciferase reporter assay. SOX12 was identified to be abnormal expressed in various cancers, such as hepatocellular carcinoma [31], CRC [32] and lung cancer [33]. SOX12 was also reported to be over-expressed and facilitated cell proliferation, migration and invasion in OS [34]. Consistently, our study revealed that SOX12 was over-expressed in OS tumor tissues. In addition, it was found that SOX12 exhibited a negative correlation with miR-874-3p in OS tissues and could be targeted by miR-874-3p in OS cells. Using a rescue experiment, we found that SOX12 restoration could neutralize the effects of miR-874-3p over-expression in OS. These results suggested that lncRNA CASC9 promoted cell invasion and proliferation by modulating SOX12 via miR-874-3p.

In conclusion, we demonstrated that CASC9 exacerbates the oncogenicity of OS cells by targeting the miR-874-3p/SOX12 axis. Our findings offered a novel insight of the therapeutic strategy in OS.

## Data Availability

All data generated and/or analyzed during this study are included in this published article.
